# The Gene Expression Profile of Peripheral Blood Mononuclear Cells from EV71-Infected Rhesus Infants and the Significance in Viral Pathogenesis

**DOI:** 10.1371/journal.pone.0083766

**Published:** 2014-01-02

**Authors:** Ying Zhang, Erxia Yang, Jing Pu, Longding Liu, Yanchun Che, Jingjing Wang, Yun Liao, Lichun Wang, Dong Ding, Ting Zhao, Na Ma, Ming Song, Xi Wang, Dong Shen, Donghong Tang, Hongtai Huang, Zhixiao Zhang, Dai Chen, Mingfei Feng, Qihan Li

**Affiliations:** 1 Yunnan Key Laboratory of Vaccine Research & Development on Severe Infectious Diseases, Institute of Medical Biology, Chinese Academy of Medicine Science and Peking Union Medical College, Kunming, China; 2 Department of Bioinformatic analyses, Novel Bioinformatics Co., Ltd, Shanghai, China; Emory University School of Medicine, United States of America

## Abstract

Enterovirus 71 (EV71) is the major pathogen responsible for fatal hand, foot and mouth disease (HFMD). Our previous work reported on an EV71-infected rhesus monkey infant model that presented with histo-pathologic changes of the central nervous system (CNS) and lungs. This study is focused on the correlated modulation of gene expression in the peripheral blood mononuclear cells (PBMCs) from EV71-infected rhesus monkey infants. The expression of more than 500 functional genes associated with multiple pathways was modulated. The expression of genes associated with immune inflammatory responses was up-regulated during the period from days 4 to 10 post-infection. The expression of two genes (TAC1 and IL17A), which play major roles in inflammatory reactions, was remarkably up-regulated during the infection period. Furthermore, a higher expression level of the TAC1 gene was identified in the CNS compared to the lungs, but a high expression level of the IL-17A gene was observed in the lungs and not in the CNS. The results of this study suggest at least two facts about EV71 infection, which are that: the TAC1 gene that encodes substance P and neurokinin-A is present in both PBMCs and the hypothalamus; and the up-regulation of IL-17A is sustained in the peripheral blood.

## Introduction

Pathogenic studies focusing on hand, foot and mouth disease (HFMD) and its emerging epidemics in the Asia-Pacific regions in recent years have demonstrated that enterovirus 71 (EV71) is one of the major pathogens responsible for human cases of HFMD, and infection with this virus occasionally leads to severe diseases and death [1], [2]. Furthermore, multiple clinical and pathological studies focusing on the fatal cases of HFMD suggest that the brainstem encephalitis caused by EV71 infection of the central nervous system (CNS) and the subsequent neurogenic heart and lung failure ultimately contribute to the severe pathogenesis in these human patients [3], [4], [5]. In this context, it is presumed that the pathogenic events observed in the CNS contributed to neurogenic heart failure or pulmonary edema; these two conditions are frequently identified as abnormal pathophysiological features triggered by the over-activation of the sympathetic nervous system [6], [7]. To date, the mechanism of this pathogenic damage process remains largely unknown. One hypothesis, based on clinical manifestations and animal experiments, is that the inflammatory response, which is characterized by a cytokine storm, is induced by EV71 infection and subsequently leads to the pathological changes in the CNS tissues, resulting in further disruption of the CNS stability [5], [8]. This hypothesis is supported by evidence that there is elevation of pro-inflammatory factors in the sera or cerebrospinal fluid of fatal cases observed in clinical manifestations and multiple animal experiments [5], [8], [9], [10]. However, whether the up-regulation of a certain type of pro-inflammatory factor or the abnormal expression of a specific pro-inflammatory factor can trigger such a pathophysiological response needs to be explored further.

In our previous studies, the basic EV71 pathogenic process was successfully mimicked in rhesus monkey infant [11].These monkeys exhibited the typical clinical manifestations, such as vesicles on the mucosa of the palate, tongue and limbs; fever;and the histopathological manifestations, such as a variety of viral loads, antigen expression and perivascular infiltration in the CNS [3], [11]. While such a pathogenic process did not result in severe neurogenic heart failure or pulmonary edema, there was remarkable alveolar inflammatory effusion and partial destruction of the alveolar wall, among other pathological changes [3], [11]. The histopathological and inflammatory pathological changes most likely suggest the presence of an inflammatory response during the pathogenic process elicited by EV71 infection [3], [11], [12]. Thus, further study of such a potential inflammatory response would be helpful in understanding its correlated pathogenic mechanism.

Microaary analysis can harness the systematic gene expression profile and its variability between tissues or cells with specific response states [13]. Based on the presumption that the human immune response is potentially involved in the pathogenic process of EV71 infection, the gene expression profile of peripheral blood mononuclear cells (PBMCs) from EV71-infected rhesus infants was comprehensively and systematically compared and analyzed at different time points after infection. The variety in the gene expression profile of PBMCs, which are major components of the immune system, was shown to contribute to individual processes and to the characterization of the immune response [14].

Our results showed a marked variety of gene expression profiles in the PBMCs of EV71-infected rhesus infants during the pathogenic process, and these profiles were characterized by the activation of the integral functions of the immune system, as well as the up-regulation of genes associated with the inflammatory response. Specifically, there was up-regulation of genes involved in the stress response and genes encoding cytokines, such as substance P and IL-17, which play major roles in allergic reactions. Certainly, the presence of these functional molecules in the major organs and tissues targeted by viral infection suggests their potential significance in the pathogenic process of EV71 infection.

## Methods

### Virus and cells

The EV71 virus (sub-genotype C4) was originated from an epidemic in Fuyang, China, 2008 (GenBank: EU812515.1) [15]. The virus was grown in Vero cells (ATCC, Manassas, VA, USA) and harvested for freezing at −20°C [3]. The Vero cells were maintained in DMEM (HyClone, Logan, UT, USA) with 10% FBS (Gibco, Grand Island, NY, USA).

### Ethics statement

The animal experiments were designed based upon the principles expressed in the “Guide for the Care and Use of Laboratory Animals” by the National Research Council of the National Academies [16] and “The Guidance to Experimental Animal Welfare and Ethical Treatment” by The Ministry of Science and Technology of the People's Republic of China [17]. The experimental protocols were reviewed and approved by the Yunnan Provincial Experimental Animal Management Association (Approval number: SCXK (Dian) 2011-0005), as well as the Experimental Animal Ethics Committee of the Institute (Approval number: YISHENGLUNZI [2011] 15).

The animals were bred in cages separately in a large room (BSL-2 conditions) with sufficient fresh air and natural light, which allowed for visual, olfactory and auditory interactions with other monkeys. The room temperature was maintained at approximately 25°C during the experiments. Food and water were readily available. Appropriate treats and vitamins were provided. The animals were given access to environmental enrichment (such as approved toys) to promote psychological well-being. All animals were fully under the care of veterinarians at Institute of Medical Biology (IMB), Chinese Academy of Medicine Science (CAMS). The housing conditions, experimental procedures and animal welfare were in accordance with the local laws and guides on the use of laboratory non-human primates and complied with the recommendations of the Weatherall report.

### Study design in monkeys

Two weeks before the infection, all animals were tested for the presence of anti-EV71 antibodies in the serum. Nine health rhesus monkeys infants (40 to 50-days-old) with weights ranging from 250 g to 350 g were divided into the following two groups: 6 in the experimental group and 3 in the negative control group. All infants were kept with their own mothers in single stainless steel cages during the entire experimental period. The mothers were fed pellets (IMB, CAMS, China), peanuts and fresh fruits.

Prior to EV71 infection, 1.5–2.0 ml of venous blood and 0.2 ml of cerebrospinal fluids (CSF) were sampled from each animal in both groups as a baseline control; serum from 0.3–0.5 ml of the blood was isolated for immunological assays, and RNA was extracted from the PBMCs that were isolated from 1–1.5 ml of blood for gene microarray assays. EV71 infection (10^4.5^ CCID_50_/animal) via nasal spray of the experimental group was performed 3 days after the daily examination [11]. PBS was administered in parallel via nasal spray to the control group animals. The animals were monitored for clinical signs, and blood samples were collected under appropriate anesthesia to alleviate pain and minimize suffering, in compliance with the guidelines of IMB, CAMS, every day after infection. Following recovery from anesthesia, all animals were returned to the colony.

Two experimental animals and one control animal were euthanized via overdose with anesthesia (overdose of isoflurane and perfused immediately [<2 min], Anesthesia Breathing System; MODEL3000, Matrx, USA), at days 4, 7 and 10 post-infection (p.i.). Before proceeding, the monkey's death was verified by the absence of heartbeat, respiration and other clinical standards (e.g. papillary reflex, etc). Venous blood for isolating the PBMCs, tissues for pathological and histochemical analyses, and CSF were sampled. At the end of the study, all monkey infants in the experimental and control group were euthanized.

### Daily examination of the animals

All live animals were monitored daily for clinical signs under anesthesia twice a day. Body temperature was measured by inserting a soft probe attached to an Omron electronic digital stick thermometer (MC-BOMR, Omron Co.) into the rectum 2 cm from the anal margin for one minute [18]. All clinical signs were monitored from day 2 p.i.. Venous blood of biological indicators such as blood cell count using the Veterinary Multi-species Hematology System (Hemavet 950FS, Drew Scientific Co.) and evaluation of the viral load were performed.

### Flow cytometry-based cytometric bead array analysis (CBA)

The serum and CSF from the infected monkeys were collected for analysis in detection assays targeting IL-2, IL-4, IL-5, IL-6, TNF-α and IFN-γ. These assays were conducted simultaneously using a Th1/Th2 cytokine cytometric bead array kit (BD Biosciences, San Diego, CA, USA) [19]. Briefly, a mixture of the anti-cytokine beads was added to the serum or CSF samples, and these samples were incubated with the included PE detection reagent in the dark at room temperature for 3 hours and then washed twice. The intensity of the resulting fluorescence signal was measured using a fluorescence-activated cell sorter (FACS) flow cytometer (FACSCanto II, BD Biosciences) and analyzed using CBA software (BD Biosciences).

### Histopathological examination of the experimental animals

Various tissue samples from the organs were fixed in 10% formalin in PBS, dehydrated in ethanol gradients and embedded in paraffin for further H-E staining [3]. The histopathological detection was performed under a light microscope.

### Immunohistochemical examination

For the immunohistochemical analysis, the tissue samples were embedded in an optimal cutting temperature (OCT) compound (Miles Inc., Elkhart, Ind.) and frozen in liquid nitrogen. The frozen tissues were then cut into 4 µm sections, placed on poly-L-lysine-coated glass slides and fixed in 3.7% paraformaldehyde. The endogenous peroxidase activity of the tissues was inhibited by treatment with hydrogen peroxide (2.5%). Substance P and IL-17 were detected using a mouse anti-substance P monoclonal antibody (Abcam, Cambridge, UK), anti-IL17A antibody (eBioscience, San Diego, CA, USA) and horseradish peroxidase (HRP)-conjugated anti-mouse IgG antibodies (Sigma, Deisenhofen, Germany) followed by color development with diaminobenzidine to detect the antigen-antibody reaction.

### Extraction of the total RNA and quantitative RT-PCR amplification

Total RNA was extracted from fresh tissue (lung, thalamus and spinal cord) homogenate from the experimental animals using a Qiagen RNeasy Mini Kit according to the manufacturer's protocol (Qiagen, Hilden, Germany). The total RNA was eluted in a final volume of 20 µl. The primers are listed in Table S1. For quantification, a single-tube RT-PCR assay was performed using the TaqMan one-step RT-PCR Master Mix in a 7500 Fast Real-time RT-PCR system (Applied Biosystems, Foster City, CA, USA). The following protocol was used for all PCR assays: 5 min at 42°C and 10 s at 95°C, followed by 40 cycles at 95°C for 5 s and 60°C for 30 s. The transcript of the conserved housekeeping gene GAPDH was used to normalize the samples because the GAPDH expression level is relatively constant.

### RNA extraction from the PBMCs obtained from the experimental group animals

PBMCs were isolated from whole blood by density gradient centrifugation over Lymphoprep (Ficoll-Paque PREMIUM; GE Healthcare, Piscataway, NJ, USA). The total RNA was collected from the PBMCs using TRIzol Reagent (Invitrogen, CA, USA) and purified using an RNeasy Mini Kit (QIAGEN, GmBH, Germany). The RNA Integrity Number (RIN) was also evaluated to inspect the RNA integrity using an Agilent Bioanalyzer 2100 (Agilent, CA, USA). The extracted RNA was temporarily frozen in 95% ethanol until further testing.

### Microarray assay

The Whole Rhesus Monkey Genome Microarray (G2519F-026806, GPL16026, Agilent, CA, USA) was chosen to screen for gene expression in the monkey PBMCs. GeneChip microarray experiments were conducted at the National Engineering Center for Biochip in Shanghai, China, according to the procedures in the Agilent technical manual. Briefly, mRNA purified from total RNA after the removal of rRNA was amplified and transcribed into fluorescent cRNA using the Low Input Quick Amp Labeling according to the manufacturer's protocol (Agilent). Labeled cRNA was purified using the RNeasy Mini Kit (QIAGEN, GmBH, Germany). Each slide was hybridized with 1.65 µl of Cy3-labeled cRNA using the Gene Expression Hybridization Kit (Agilent) in a Hybridization Oven (Agilent). After 17 hours of hybridization, slides were washed in staining dishes (Thermo, MA, USA) with the Gene Expression Wash Buffer Kit (Agilent). Slides were scanned using an Agilent Microarray Scanner (Agilent), and the raw data were obtained using the Feature Extraction Software 10.7 (Agilent) and normalized using the quantile algorithm with Gene Spring 11.0 (Agilent). The systemic bioinformatic analyses of microarrays test were processed by Novel Bioinformatics Co., Ltd (Shanghai, China). Briefly, the normalization value was set to 1. The differentially expressed genes, with fold change ≥ 2 or <0.5, were analyzed. Significant enrichment pathway were determined from these differentially expressed genes using the KEGG database, then the KEGG database was used to build the network of genes according to the relationship among the genes, proteins and compounds in the database [20], [21], [22], [23]. The raw microarray data were submitted to the Gene Expression Omnibus database and are available under the accession number GSE51103.

### Neutralizing antibody assay

The EV71 titer was analyzed by performing a microtitration assay using a standard protocol [24]. A mixture of diluted serum containing anti-EV71 antibodies and the virus at a titer of 500–1000 CCID_50_ in 100 µl PBS was incubated at 37°C for 1 h. The cellular pathogenic effect (CPE) of the virus was examined by inoculating the mixture onto Vero cells grown in 96-well plates [25].

### Statistical analysis

The data of the various detections are expressed as the mean values with standard deviations. Individual detection was performed in triplicate. GraphPad Prism software (San Diego, CA, USA) was used for the statistical analyses. The differences between the two groups were evaluated using one-way ANOVA. A P-value of <0.05 was considered significant.

## Results

### The EV71 infection process is associated with the modulation of gene expression in PBMCs

Our previous studies showed that EV71 infection of rhesus monkeys, specifically monkey infants via the respiratory tract, could directly lead to the rapid development of clinical manifestations, as observed by pathological examination (Fig. S1), and the pathological manifestations were identical to those previously reported [11]. In this study, the PBMCs were isolated from the infected animals at 4, 7, and 10 days post infection (p.i.), and the gene expression profiles of these cells were determined using microarray analysis. More than 1000 genes were up- or down-regulated to varying degrees (Table S2).

**Figure 1:**
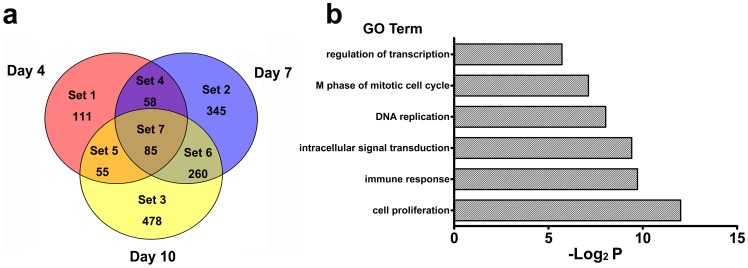
EV71 infection induced changes in the gene expression in PBMCs from infected rhesus infants. (a) A global view of the gene modulation in the PBMCs of infected infants at days 4, 7 and 10 p.i., is shown as a Venn Diagram. The colors indicate the number of significantly modulated genes at day 4 (red), 7 (purple) and 10 p.i. (yellow). Different sets show the number of modulated genes at different time point. Set 1: the modulated genes at day 4 p.i.; Set 2: at day 7 p.i.; Set 3: at day 10 p.i.; Set 4: at days 4 and 7 p.i.; Set 5: at days 4 and 10 p.i.; Set 6: at days 7 and 10 p.i.; Set 7: at days 4, 7 and 10 p.i.. (N = 6 in experimental group; N = 3 in control group). (b) Distribution of GO term identified in comparisons of monkeys infected with and without EV71. Gene ontology analysis of the differential genes in the PBMCs from infected and un-infected infants during 4, 7 and 10 days p.i.. The values are shown on a log_2_ P-value scale.

Among these genes, the expression of 85 genes was altered at all 3 time points tested, while the expression of 58 and 260 genes changed between 4 and 7 days p.i. and between 7 and 10 days p.i., respectively. Additionally, the expression of 55 genes that varied on day 4 p.i. was restored to their original levels on day 7 p.i. and varied again on day 10 p.i. (Fig. 1a). The genes separated into 6 categories, with variable distribution over time, especially for those primarily associated with transcriptional regulation and the immune response (Fig. 1b).

To confirm the results of the microarray analysis, 5 individual genes were randomly selected from each functional category and were confirmed by qRT-PCR quantitation (Fig. S2). Collectively these results suggest clear changes in the gene expression profile of PBMCs, which are frequently defined as an essential component of the immune system, and these changes are most likely linked to the clinical manifestations that develop during the progression of viral infection. Additionally, the observation that some genes culminate at day 10 when the pathological process approaches the end also implies that the pathogenesis is not dependent on the immune system, which requires further investigation.

### The immune characterization of the gene expression in PBMCs from EV71-infected rhesus infants

Because PBMCs are the identified functional vectors for natural and specific immune responses [14], they frequently display compositional variety in their cellular populations and activation of functional system-states during the viral infectious process [14].These corresponding systematic responses often reflect the generalized immune response [14]. In the current study, routine blood measurements indicated a clear increase in the lymphocyte population, similar to other viral infections (Fig. S3), as well as slight increases in the numbers of eosinophils and basophils (Fig. S3) in PBMC at different time points after inoculation with EV71.

**Figure 2:**
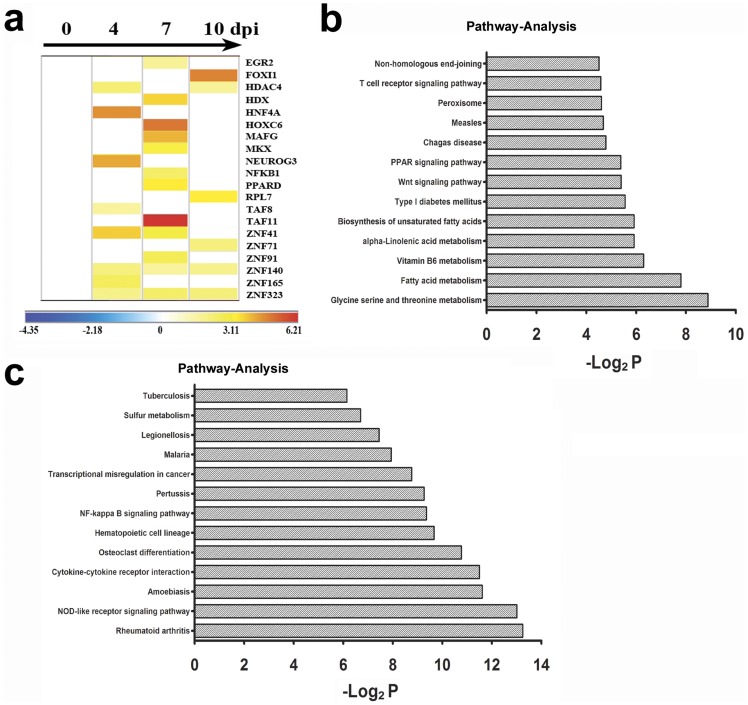
The characterization of the gene expression profiles of the PBMCs from EV71-infected rhesus infants. (a) The expression heat map of important genes associated with transcriptional regulation during EV71 infection. (b) The up-regulated expression of important genes associated with cellular metabolism, structure and signal transmission during EV71 infection. (c) The down-regulated expression of important genes associated with cellular metabolism, structure and signal transmission during EV71 infection. The color scale indicates the levels of gene expression from low (green, blue and purple) to high (red, pink and yellow). The values are shown on a log_2_ scale.

Additionally, the analysis of the gene expression profiles of the PBMCs at different time points showed a marked up-regulation of over 20 genes associated with cellular transcriptional regulation (Fig. 2a); for example, TAF11, a component of the TBP complex that plays a critical role in the binding of the transcriptional complex to the TATA motif of promoters was up-regulated 74-fold compared to the baseline [26]. Concomitantly, the expression of genes associated with cellular metabolism and structure and the majority of the cellular signal transmission genes, including proteinase and receptor genes, wasremarkably up-regulated (Fig. 2b). In contrast, the expression of genes associated with the NOD-like receptor or the NF-kappa B signaling pathway was gradually down-regulated (Fig. 2c). These data suggest that there is a clear activation response elicited from the immune response system during the EV71 infectious process. Nevertheless, whether such a transcriptional immune response could lead to the pathogenesis observed ater EV71 infection remains largely unknown in the light of quantitative evaluation.

### The modulation of the expression of genes involved in pro-inflammatory events in EV71-infected rhesus infants

**Figure 3:**
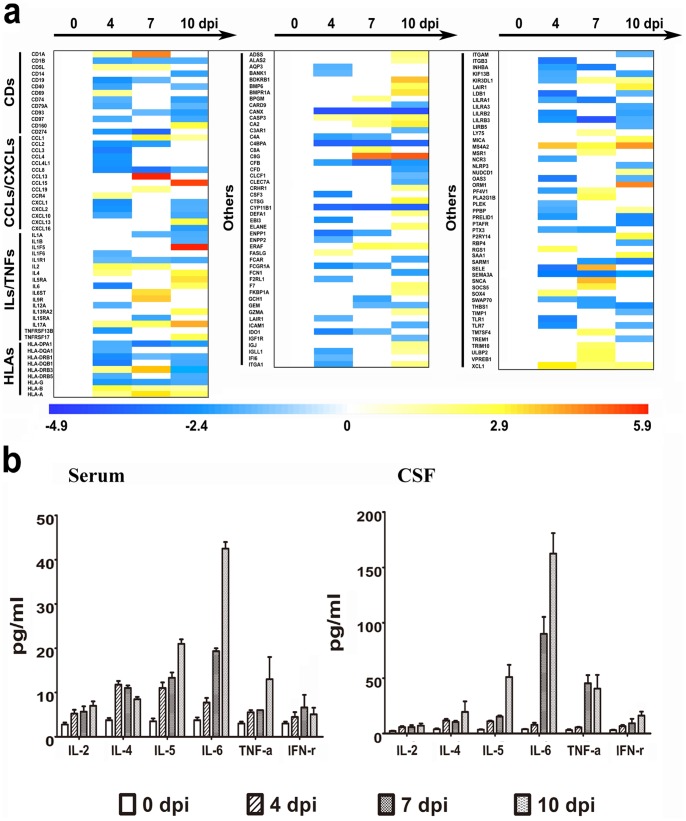
The modulation of the expression levels of genes required for pro-inflammatory events. (a) Heat map of up- and down-regulated proinflammatory cytokine genes correlated with immune responses. The color scale indicates the levels of gene expression from low (green, blue and purple) to high (red, pink and yellow). The values are shown on a log_2_ scale. (b) Quantification of proinflammatory cytokines in serum and CSFs. All cytokine (IL-2, IL-4, IL-5, IL-6, TNF-α and IFN-γ) levels were measured with a Th1/Th2 cytokine cytometric bead array kit.

As previously demonstrated during pathogenic studies of EV71 infection, the up-regulation of pro-inflammatory cytokines, such as interleukins, chemokines [5], [27], and interferon-like pro-inflammatory cytokines [5], [27], was frequently identified in the serum or cerebrospinal fluid collected from severe HFMD patients[28]. Furthermore, similar results were noted in animal models of EV71 infection [7], [11]. Thus, we wanted to analyze the gene expression profile of the associated pro-inflammatory cytokines in PBMCs from EV71-infected rhesus infant. There was a clear up-regulation in the expression of more than 50 genes associated with the immune inflammatory responses during the period of 4 to 10 days p.i. (Fig. 3a). For example, CCL13 and CCL15 both exhibited large increases in their expression levels, with CCL13 reaching a 56-fold increase on day 7 and CCL15 reaching a 42-fold increase on day 10. Additionally, the expression of interleukins, including interleukin-2 (IL-2), IL-4, and so on, tended to be up-regulated (Fig. 3a).In contrast, the IFN-like pro-inflammatory cytokines failed to exhibit up-regulation, and other partial pro-inflammatory cytokines, including CXCL1, CXCL2, CXCL10, CCL8 and IL1A, were down-regulated (Fig. 3a).

**Figure 4:**
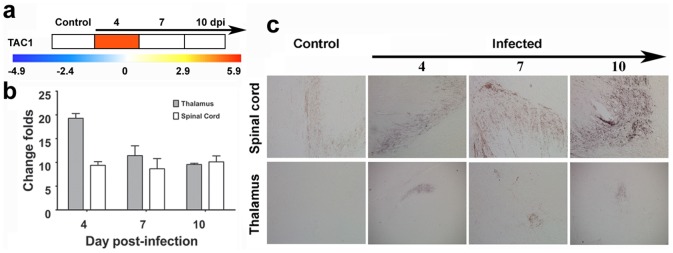
TAC1 gene expression is consistently up-regulated in the CNS from EV71-infected rhesus infants. (a) The fold-change of the TAC1 gene expression in the PBMCs from EV71-infected rhesus monkey infants according to microarray analysis. The color scale indicates the levels of gene expression from low (green, blue and purple) to high (red, pink and yellow). The values are shown on a log_2_ scale. (b) The levels of TAC1 gene expression in the thalamus and spinal cord from the EV71-infected rhesus monkey infants were measured by real-time qPCR. The y-axis indicates the relative quantity of the TAC1 mRNA in the samples compared with the control (Y = 1), and normalized to the endogenous GAPDH expression level. The samples were collected at days 4, 7 and 10 p.i.. Individual detection was performed in triplicate. Error bars are presented as the mean±SD. (c) The expression of Substance P in the thalamus and spinal cord were measured by immunohistochemical assay. The samples were tested using an anti-substance P antibody and an HRP-conjugated anti-mouse IgG antibody. Images are shown at 200× magnification.

**Figure 5:**
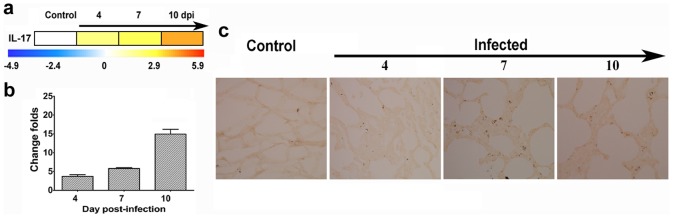
Up-regulation of the IL-17 gene in lung tissues from EV71-infected rhesus monkey infants. (a) The fold-change of IL-17 gene expression in the PBMCs from EV71-infected neonatal rhesus monkeys according to microarray analysis. The color scale indicates the levels of gene expression from low (green, blue and purple) to high (red, pink and yellow). The values are shown on a log_2_ scale. (b) The levels of IL-17 gene expression in the lungs of the EV71-infected neonatal rhesus monkeys were measured by real-time qPCR. The y-axis indicates the relative quantity of the IL-17 mRNA in the samples compared with the control (Y = 1), and normalized to the endogenous GAPDH expression level. The samples were collected at days 4, 7 and 10 p.i.. Individual detection was performed in triplicate. Error bars are presented as the mean±SD. (c) The expression of IL-17 in lung tissues was measured by an immunohistochemical assay. The samples were tested with an anti-IL17A antibody and HRP-conjugated anti-mouse IgG antibody. Images are shown at 400× magnification.

Based on this observation, we further quantified the protein levels for select cytokines in the CSF and serum of EV71-infected monkeys including IL-2, IL-4, IL-5, IL-6 and TNFα. All were up-regulated during the progression of viral infection (Fig. 3b). However, the fact that the up-regulation of cytokines was asynchronous in serum and CSF suggests a predominant pathological aspect in the CNS during EV71 infection. The expression levels of 2 particular genes were interesting. The expression of the TAC1 gene, which encodes essential stress polypeptides including substance P and neurokinin A, increased as much as 36-fold on day 4 p.i. (Fig. 4a). Additionally, the IL-17A gene has critical pro-inflammatory effects in the immune allergic reaction, and the expression of this gene increased 3.7-, 5.85- and 16.2-fold by 4, 7 and 10 days p.i., respectively (Fig. 5a). These 2 molecules have received substantial attention due to their essential functions in the CNS stress reaction and immune system, as well as for their significance in the expression of allergic reactions in humans [29], [30], [31].

### TAC1 is transiently over-expressed in PBMCs and consistently up-regulated in the CNS

The TAC1 gene has been widely recognized to play a major role in the nervous and immune systems [30], [31] and exhibits different expression patterns in response to distinctive stresses by targeting specific stimulation signals. This gene encodes substance P and neurokinin A, which functions crucial neurotransmittors and specific neuromodulators of the immune system [32]. Both of these peptides also play significant roles in neuron-inflammatory events, including the generation of blood vessel anapetia, the increase in vascular permeability and plasma protein extravasation [33]. Importantly, substance P and its functional receptor, NK1, were found to be widely distributed in the thalamus, as well as in the neuron bodies and nerve fibers [34]. In the immune system, substance P is generated by monocytes, lymphocytes and macrophages [35], [36], and stimulates the activation of these cells, as well as increases the secretion of multiple pro-inflammatory cytokines [37], [38]. Simultaneously, the up-regulation of their expression might enhance the inflammatory response. Microarray analysis of PBMCs indicated a 36-fold increase in the expression of the TAC1 gene 4 days p.i. (Fig. 4a), and the expression was subsequently restored to its original state 7 days p.i.

On the basis of these results and findings from the CNS and lungs that suffered inflammatory pathological injuries, qRT-PCR and immunohistochemical assays were performed to detect the expression of substance P in the CNS and lung tissues from EV71-infected rhesus infants at 4, 7, and 10 days p.i.. qRT-PCR analyses showed that the TAC1 gene was up-regulated 18-, 12- and 9-fold in the CNS on days 4, 7 and 10 days p.i., respective to non-infected controls (Fig. 4b). In contrast, this gene was not expressed in the lung tissues (data not shown). A histochemical examination using an anti-substance P monoclonal antibody revealed a high expression of substance P, encoded by the TAC1 gene, in the thalamus and the posterior horn of the spinal cord (Fig. 4c), whereas no substance P expression was observed in the lung tissues (data not shown). These data suggest that a product of the TAC1 gene, specifically substance P, might play a crucial role in the initiation of the pathogenic events during the progression of EV71 infection.

### IL-17A gene expression is consistently up-regulated in PBMCs

IL-17A is thought to be an essential cytokine that regulates pathogenesis in multiple infections [29]. This type of cytokine is usually produced either by the Th17 subtype of T lymphocytes, neutrophil granulocytes or by mucosal NK cells recruited to the sites of inflammation during an immune response [39], and its cell of origin is linked to its functional significance in the inflammatory response of the human body. Additionally, IL-17A can stimulate the respiratory epithelium to generate IL-8 and CXC-like chemokines, including growth-related oncogene (GRO)–α, granulocyte chemotactic protein (GCP-2) [40], [41], [42], IL-6 and G-CSF [43]. Furthermore, a study of the potential function of IL-17A in the pathological injury demonstrated that IL-17A is a crucial factor in the mechanism of the lung host defense and the associated pathogenic damage [44], [45].

As shown by the microarray results, the expression of the IL-17A gene tended to gradually increase during the progression of viral infection (Fig. 5a), which was consistent with both of the results from the qRT-PCR amplification on lung tissues (Fig. 5b) and the immunohistochemical staining of the pulmonary alveolar walls of the lung tissues (Fig. 5c). Collectively, these data suggest that IL-17A expression may have pathological significance in EV71 infection, and this effect may be attributed to its role in the immune response. No IL-17A expression was noted in the CNS tissues (data not shown).

## Discussion

Many recent studies on the pathology of EV71 infection in severe and fatal cases of HFMD have demonstrated that a number of pathological events, specifically brainstem encephalitis and the subsequent neurogenic pulmonary edema, are most likely due to the inflammatory immune response [46]. The histopathological examination and the up-regulation of pro-inflammatory cytokines in the serum and cerebrospinial fluid, including the so called pro-inflammatory cytokine storm [5], [47], suggest an abnormal immune response [46]. It has been shown that EV71 can infect the immune cells, including monocytes or immature DCs, leading to the altered expression of individual immune mediators [48], [49]. Additionally, molecular studies have demonstrated that EV71 can inhibit the expression of IFN-like molecules in cells, which in turn impacts the innate immune response via its encoded proteins, such as 3C protein [50]. This provided the rationale for testing gene expression profiles of PBMCs in the context of our EV71 infected rhesus macaque infant model [11] using microarray technology, to better detail the immune response to this infection. We hypothesized that a close correlation exists between the mechanism of EV71 pathogenicity and an abnormal immune response.

As one of the major component of the immune system, PBMCs have been shown to be hypersensitive and rapidly reactive to viral infection [14]. The significant functional genes associated with immune activity displayed in the gene expression profile of PBMCs in the EV71 infected animals imply that there is an integral systemic immune response. The up-regulation of some pro-inflammatory cytokines was observed, such as substantial increases of chemokines (CCL13, CCL13 and etc.) compared to their levels prior to infection, and this result suggesting that there is a potentially excessive immune response in the EV71 infection process. The clinical symptoms and the viral loads in the different organs of the infected animals, as well as the histopathological examination, collectively suggest the potential significance of the varied gene expression.

Based on the characterizations of the immune system during EV71 infection, there were 2 up-regulated factors involved in the inflammatory reaction and respiratory mucosal immunity that were potentially significant; these included IL-17 and TAC1. Although these two factors seem to be functionally independent, the integration of their activities in the immune response might provide a clue for understanding the EV71 pathogenesis. Previous studies have suggested that tachykinins, including substance P and neurokinin A as the important neuropeptides, can potentiate cholinergic neurotransmission in the CNS and postganglionic nerve terminals [51], [52]. This neuropeptides was also synthesized in non-neuronal cells, such as macrophages and released in inflammatory diseases [35]. Because their receptors NK1 (for SP) and NK2 (for NKA) were located in the epithelial cells, vessels, submucosal glands and smooth muscle of respiratory system, tachykinins most likely showed a more important constricting effect on the smaller bronchi and microvascular of the bronchi and alveoli in asthmatic subjects [53], [54], [55], [56].

As an effector cytokine, IL-17 also plays a crucial role in the inflammatory processes, which could lead to autoimmunity and host defense [57]. IL-17 was also capable of recruiting neutrophils to inflammatory tissue through stimulating IL-8 and MIP-2 production in epithelial cells [58]. Both factors showed activity that impacts the respiratory physiological process and most likely results in lung and bronchial disorders, which are typical pathophysiological events that are clinically observed in severe case of EV71 infection [59], [60]. In this case, it is reasonable to hypothesize that the significant up-regulations of the TAC1 and IL-17 genes might be related to EV71 pathogenesis, especially with respect to the pathological outcome induced by EV71 infection in the CNS. Interestingly, based on the up-regulation of the TAC1 gene in PBMCs, histochemical examination performed over the same time period demonstrated that the expression of substance P in the hypothalamus of EV71-infected animals was remarkably higher than in un-infected animals.

The existing data suggests that TAC1 is involved in the central control of peripheral autonomic functions, such as blood pressure and the rate of respiration [61], while its functioning as an inflammatory agent in the immune reaction induced by EV71 observed here. Furthermore, the sustained up-regulation of the IL-17A gene in PBMCs during EV71 infection also suggests that IL17A, as a cytokine responsible for allergic diseases of the respiratory tract, could play an important role in the activation of the immune inflammatory effect through stimulating some of the inflammatory factors and neutrophil recruitment [58]. Similarly, as confirmed by histochemical examination and RT-PCR, the expression level of IL-17A in the lungs of EV71-infected animals exhibited a sustained increase compared with the control group. Based on the fact that IL-17A was produced by innate immune cells, the increasing expression of this gene in the lung during pathogenesis implies a positive feedback cycle in which the allergic effects could be amplified by the increased neutrophil recruitment by IL-17A, exacerbating further the increase in the secretion of IL-17A by the recruited neutrophils. Substance P can improve the production of Th17 cells by targeting the immune system [62]. Strikingly, the dynamic up-regulation of substance P in the PBMC and CNS appears to be correlated with the increase of IL-17A in the lungs, an organ damaged in the pathogenesis of EV71 infection. Whether this relationship can directly lead to the elucidation of the mechanism behind the pathological changes during EV71 infection is an interesting issue worthy of further exploration.

Study limitations, access to various tissues to study molecular mechanism of the pathogenesis required euthanasia of subjects, therefore limiting our ability to harness the dynamic profiles of the TAC1 and IL-17A gene expression. Nevertheless, the results of our current study provide new insight that warrant further detailed investigation of the pathomechanisms of EV71 infection.

## Supporting Information

Figure S1 Clinical manifestations and pathological lesions in the EV71-infected rhesus infants. (a) Vesicular lesions (arrow) in the mouth and feet of a rhesus monkey infant. (b) Body temperature monitoring of the EV71-infected rhesus monkeys. The body temperatures of the infected monkeys were measured via the rectal route twice each day post-infection. The normal body temperatures of the controls are shown as a dotted line. Bars represent the mean ± SD. (N = 6 in experimental group; N = 3 in control group). (c) Viral RNA which was collected on day 1 to 10 post-infection, was extracted from blood specimens and measured with a real-time qPCR assay. Bars represent the mean ± SD. (N = 6 in experimental group; N = 3 in control group). (d) Pathological changes in the target organs (lungs and thalamus) from infected neonatal rhesus monkeys on days 4, 7 and 10 p.i.. Infiltration of inflammatory cells (black arrow), edema and hemorrhage (blue arrow). Images are shown at 200× magnification.
(TIF)

Figure S2 Confirmation of the gene expression changes using qRT-PCR. Five individual genes were random selected from each functional category and were analyzed using qRT-PCR. The y-axis indicates the relative quantity of the specific mRNA in the samples compared with the control samples. The results are normalized to the level of endogenous GAPDH expression. Individual detection was performed in triplicate. Error bars are presented as the mean±SD.
(TIF)

Figure S3 Blood cell analysis of the PBMCs collected from the EV71-infected rhesus infants at 4, 7, and 10 days p.i. Venous blood was collected for the routine analysis of biological indicators at 0, 4, 7 and 10 days p.i. The bars represent the maximum and minimum. The mean of the percentage of cells with a 95% CI is shown as the rectangle. The line in the rectangle represents the mean of the percentage of cells.
(TIF)

Table S1 Sequences of primers for real-time RT-PCR amplification of 59 selected genes.
(DOC)

Table S2 Differentially expressed genes in rhesus neonates during the EV71 infection by microarray assay.
(DOC)
