# Supplementary figures and images for: The Gene Expression Profile of Peripheral Blood Mononuclear Cells from EV71-Infected Rhesus Infants and the Significance in Viral Pathogenesis
Source: PLoS One. 2014 Jan 2;9(1):e83766. doi: 10.1371/journal.pone.0083766 (PMC3879270; doi:10.1371/journal.pone.0083766)

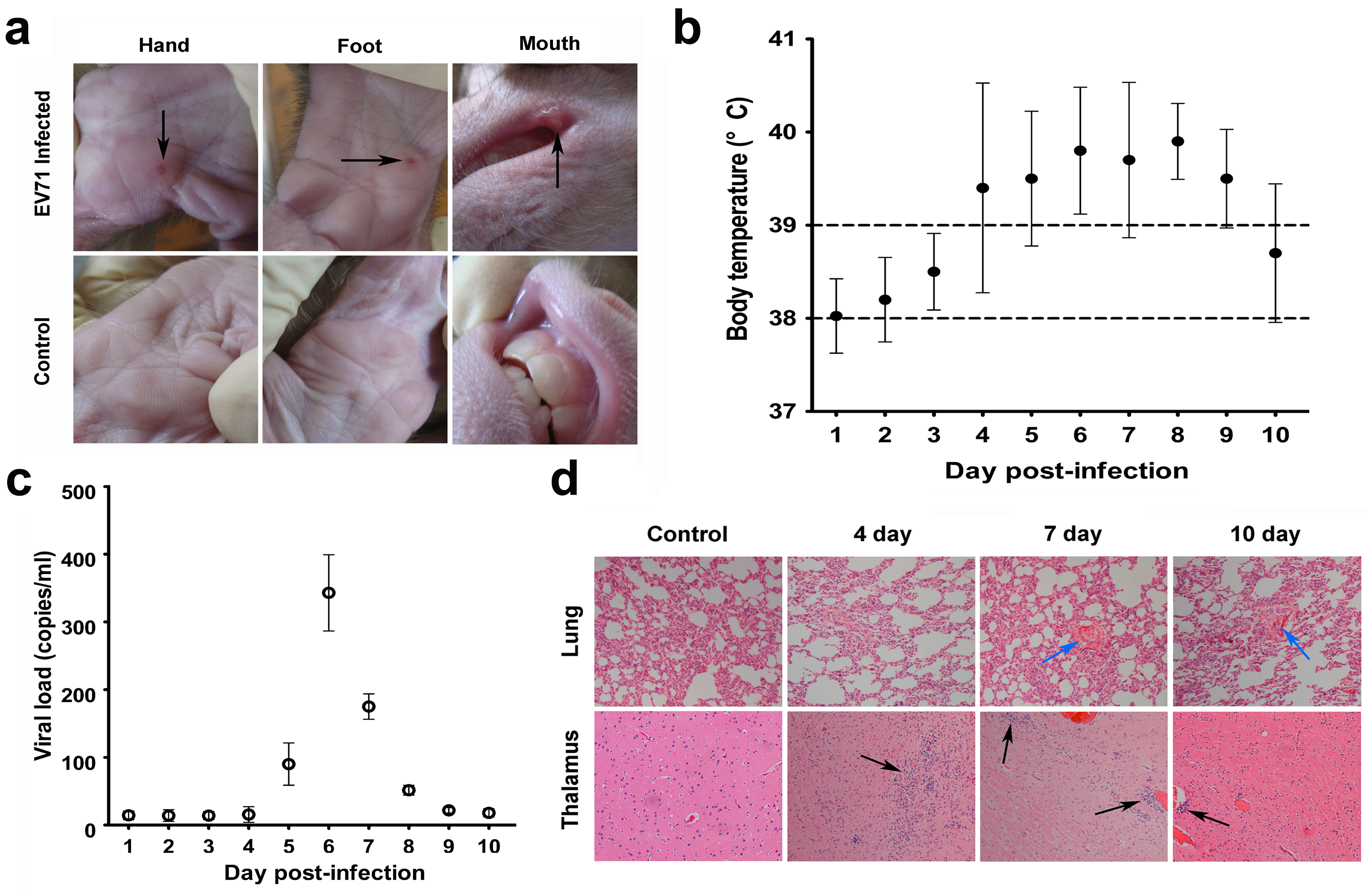

Supplement: Figure S1 — Clinical manifestations and pathological lesions in the EV71-infected rhesus infants. (a) Vesicular lesions (arrow) in the mouth and feet of a rhesus monkey infant. (b) Body temperature monitoring of the EV71-infected rhesus monkeys. The body temperatures of the infected monkeys were measured via the rectal route twice each day post-infection. The normal body temperatures of the controls are shown as a dotted line. Bars represent the mean ± SD. (N = 6 in experimental group; N = 3 in control group). (c) Viral RNA which was collected on day 1 to 10 post-infection, was extracted from blood specimens and measured with a real-time qPCR assay. Bars represent the mean ± SD. (N = 6 in experimental group; N = 3 in control group). (d) Pathological changes in the target organs (lungs and thalamus) from infected neonatal rhesus monkeys on days 4, 7 and 10 p.i.. Infiltration of inflammatory cells (black arrow), edema and hemorrhage (blue arrow). Images are shown at 200× magnification. (TIF) [file pone.0083766.s001.tif]

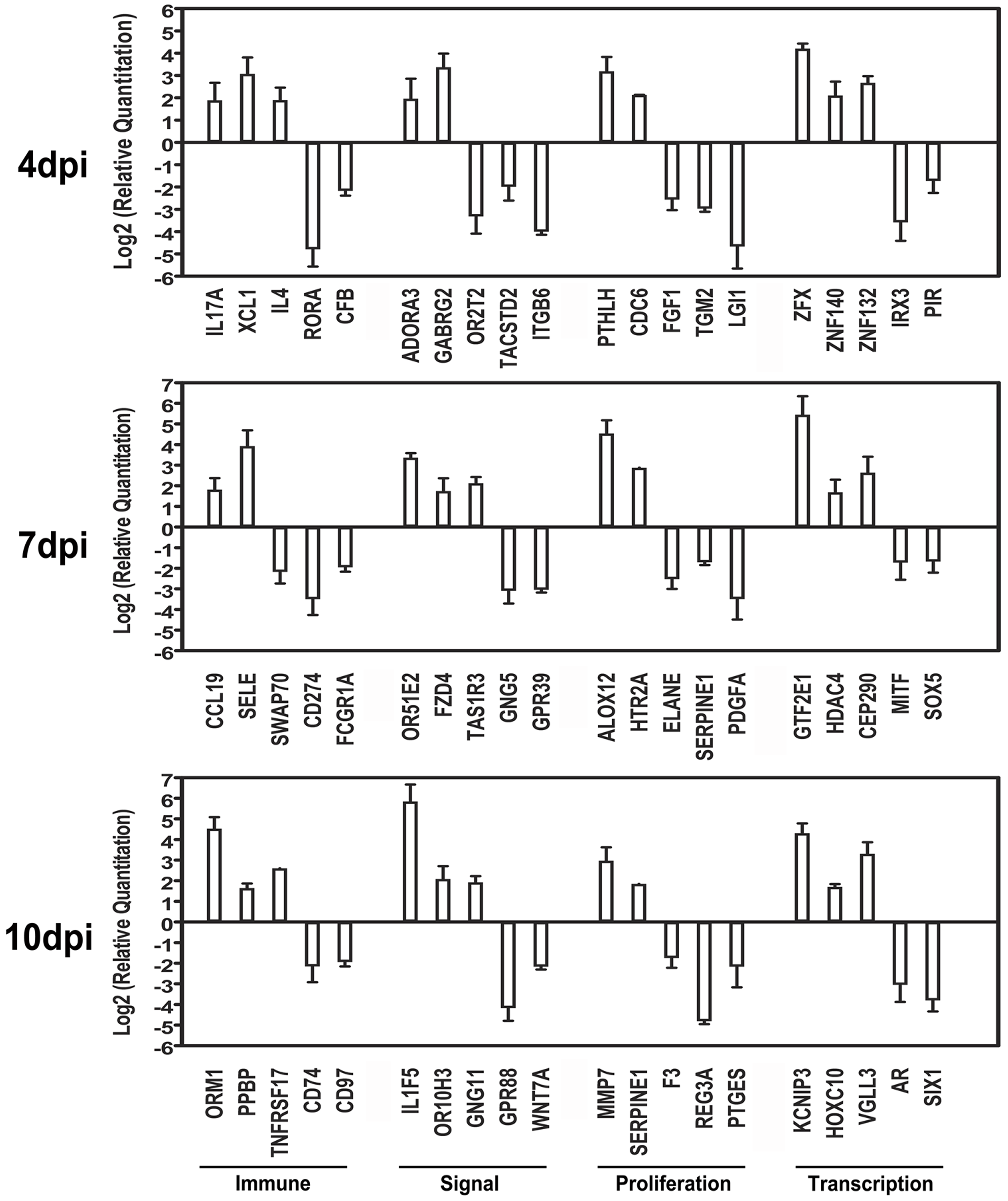

Supplement: Figure S2 — Confirmation of the gene expression changes using qRT-PCR. Five individual genes were random selected from each functional category and were analyzed using qRT-PCR. The y-axis indicates the relative quantity of the specific mRNA in the samples compared with the control samples. The results are normalized to the level of endogenous GAPDH expression. Individual detection was performed in triplicate. Error bars are presented as the mean±SD. (TIF) [file pone.0083766.s002.tif]
