# Supplementary material for: The Gene Expression Profile of Peripheral Blood Mononuclear Cells from EV71-Infected Rhesus Infants and the Significance in Viral Pathogenesis
Source: PLoS One. 2014 Jan 2;9(1):e83766. doi: 10.1371/journal.pone.0083766 (PMC3879270; doi:10.1371/journal.pone.0083766)
Supplement: Table S1 — Sequences of primers for real-time RT-PCR amplification of 59 selected genes. (DOC) [file pone.0083766.s004.doc]

**Table S1** Sequences of primers for real-time RT-PCR amplification of 59 selected genes.

| **Classical** | **Gene name** | **Primer sequence (5’→3’)** | | **Target size (bp)** |
| --- | --- | --- | --- | --- |
|  |  | **Sense** | **Anti-sense** |  |
| **Immune response** | IL17A | TCAACGCTGATGGGAACGT | GCGCAGGACCAGGATCTCT | 71 |
|  | XCL1 | GGCTCCTTGAGAGCAGTAATTTTT | TCTCACCCATGTGGCTTGTG | 78 |
|  | IL4 | CACAGGCACAAGCAGCTGAT | GCCAGGCCCCAGAGGTT | 62 |
|  | RORA | TGTTTGATTGATCGAACCAGTAGAA | ATCCCTACGGCAAGGCATT | 71 |
|  | CFB | CCACTGCTATGACGGTTACACTCT | CACTCCATCGGCCATTCACT | 71 |
|  | CCL19 | GCCTGCTGGTTCTCTGGACTT | CAGCAGTCTTCAGCATCATTGG | 67 |
|  | SELE | ACCTCCACGGAAGCTATGACTTAT | TGAATTGCAACCAGGTGTGTGT | 77 |
|  | SWAP70 | AGCCATTCATTCTACTATTCATCTGTTG | CGCTGGCGGGCTTCTT | 69 |
|  | CD274 | GCTGAATTGGTCATCCCAGAA | GGCTCCCAGAATTACCAAGTGA | 75 |
|  | FCGR1A | GAATCACCTCTGCCAGTGTCAAT | GGGTCACTTCGCCCTGAGA | 73 |
|  | ORM1 | AGCCTCCTACCTCTGCTGGAA | GGTGATGGGCACCGGTACT | 66 |
|  | PPBP | CCTCTGGAATTCATCCCAAAAA | ACTTGGTTGCAATGGGTTCCT | 70 |
|  | TNFRSF17 | AGCAAACCGAAGGTCGACTCT | GGTTGCGCCTTCCTCCAT | 63 |
|  | CD74 | GAGCTGTCGGGAAGATCAGAAG | GGCAGTTGCTCATTGTTGGA | 72 |
|  | CD97 | GCCACAACAACACCAAGGAACT | TCCCCATCGGAGGACTCA | 73 |
|  |  |  |  |  |
| **Cell proliferation** | PTHLH | CCTCGGAGGTGTCCCCTAAC | CCCTCATCATCAGACCCAAATC | 79 |
|  | CDC6 | CCGTAACCTGTTCTCCTCGTGTA | GTTGTCATCGCCCAGACGTT | 68 |
|  | FGF1 | GGACACCGACGGGCTTT | CTCCAGCCTTTCCAGGAACA | 70 |
|  | TGM2 | TCAATGCCGACGTGGTAGAC | TCAGGGAACGGTTGATGGAT | 72 |
|  | LGI1 | GCCACACCAATGCAACTGTT | TATTGATTTTGCGCTTCTTGTATTCT | 72 |
|  | ALOX12 | CAGGACTGCACCCCATCTTC | CCCGGGTGTTGATTTCCA | 69 |
|  | HTR2A | CCTCTGCCGAGCAAGCTTT | GATGGAGGCCGTGGAGAA | 66 |
|  | ELANE | CCACCCGGCAGGTGTTC | GTGGCCGACCCGTTGAG | 103 |
|  | SERPINE1 | ATCAATGACTGGGTGAAGACACA | TGGTCCACGGCTCCTTTC | 71 |
|  | PDGFA | AAGGTGGCCAAGGTGGAATAC | GCAGGCGCACTCCAAATG | 87 |
|  | MMP7 | CTGTATGCTGCAACTCATGAACTTG | TGGATACATCACTGCATTAGGATCA | 78 |
|  | F3 | GAACCCAAACCCGTCAATCA | TGTCTGTTGTGTAAAAGCATTTGC | 88 |
|  | REG3A | GGGCTGAGGGATCCTTCGT | AGCCCAATCCAGACGTATGAGT | 73 |
|  | PTGES | GTCTACTCCTTTCTGGGTCCTAACC | ACGGCCCACGAGGAAGA | 69 |
|  |  |  |  |  |
| **Transcription** | ZFX | TGAATTACAACAAGAGCCAAACTCA | AATTTGATCACCATCCATGTGTGT | 76 |
|  | ZNF140 | AAGGCCCTGTGTATTCCAGTTTT | CCCTGATTTTCTTGCAGCATCT | 76 |
|  | ZNF132 | ATGCAGATCCTTCCACCAAGA | ATGCTCAGCCAGGTGCAAA | 83 |
|  | IRX3 | CCAAAAATAGCGACTCGGAAGAT | GCCAGACTCAGGACCGGTAGT | 70 |
|  | PIR | CAACCTTATATTTGGACTTCAAATTGG | AAATGAAGCTTGTCCACCCTTTAG | 81 |
|  | GTF2E1 | GAATCAGCAATGCCCAAAAAA | GCATAAATGGGCTCAATTTGTTC | 74 |
|  | HDAC4 | GGCCAGCACAGAAGTGAAGAT | TGGAAATGCAGTGGTTCAGATT | 92 |
|  | CEP290 | CAAACAAACGGCTTGGGATTT | TTTGACCAAGAGTGAGGAAAGAGA | 133 |
|  | MITF | CGAGCTCATGGACTTTCCCTTA | TGATGATCCGATTCACCAAATC | 70 |
|  | SOX5 | GGCGCAAGGGCAGTTTAG | TTTGATGAGCTCTTCCATTTTCC | 68 |
|  | KCNIP3 | TCAAACTCATTTACGCGCAGTT | CGCATCAAAGGCGTTGAAG | 80 |
|  | HOXC10 | AGGAAATTGGCTGACAGCAAA | CAATTCCAGCGTCTGGTGTTT | 73 |
|  | VGLL3 | TGCTGATGCCTTCAGTGCAT | ACTGTAGTTGGTTCTGTCTTTGTGATGT | 76 |
|  | AR | TGTCAACTCCAGGATGCTCTACTT | GCTGTACATCCGGGACTTGTG | 79 |
|  | SIX1 | CCACCGCGGCAACTTC | GGTGGTTGTGAGGCGAGAA | 68 |
|  |  |  |  |  |
| **Signal Transduction** | ADORA3 | TTTTCATCCCCCTGGTTGTC | CTGAGTTTGTTCCGAATGATGTAAA | 70 |
|  | GABRG2 | GGAAATCGCTCCCCAAGGT | GAAGACAAAGATGAAACAAACAGATACA | 74 |
|  | OR2T2 | GCAGGGTTTGCTTATTCATGGT | AGCATGAACCCATCCAAGGA | 64 |
|  | TACSTD2 | CCTACTACTTCGAGAGGGACATCAA | AGGCGCTTCATGGAGAACTTC | 151 |
|  | ITGB6 | ATTGCGCCTCAAAGCTTGA | CAGTCTGGCGGACATGCA | 76 |
|  | OR51E2 | GCTGCTGCCTCCTGTCATC | AGCACCCGTGTTCTGATCTGT | 69 |
|  | IL1F5 | CGGCATTGAAGGTGCTTTATC | TGACCTTCCCTGCATGCA | 69 |
|  | FZD4 | TGGGTGAAAAGGAGCCTGAA | CTTGGCTGAGCGGCTGTATAA | 72 |
|  | TAS1R3 | CCGCAGTGTGACTGCATCAC | CACGCTATACACAGCTGCGTAGA | 90 |
|  | GNG5 | CAGCTGCAGACTTGAAACAGTTCT | TCTGAAGGGATTTGTACTTGAAGATACT | 89 |
|  | GPR39 | CCCTTCTCGGAGACGTTTTTCT | TGCGAGGACACCGTGTACA | 68 |
|  | OR10H3 | TGGCCACAGTTTGGATTGAAC | CAGAGATGGAGAGGGCACACA | 72 |
|  | GNG11 | TGCCAGAGAAGGAAAAACTGAAA | CATTTAGACACTTGTTGTCTCTGCAA | 82 |
|  | GPR88 | ATCCCGGTGTCACTCCTGTATT | TCGGAAGGACGACACGAGAT | 87 |
|  | WNT7A | TCGCCAAGGTCTTTGTGGAT | CGTTGTTGTGCAAGTTCATGAGA | 72 |
