# Supplementary material for: The Gene Expression Profile of Peripheral Blood Mononuclear Cells from EV71-Infected Rhesus Infants and the Significance in Viral Pathogenesis
Source: PLoS One. 2014 Jan 2;9(1):e83766. doi: 10.1371/journal.pone.0083766 (PMC3879270; doi:10.1371/journal.pone.0083766)
Supplement: Table S2 — Differentially expressed genes in rhesus neonates during the EV71 infection by microarray assay. (DOC) [file pone.0083766.s005.doc]

**Table S2** Differentially expressed genes in rhesus neonates during the EV71 infection by microarray assay.

| **Classes** | **Symbol** | **Gene Name** | **Change Folds** | | |
| --- | --- | --- | --- | --- | --- |
|  |  |  | **4 dpi** | **7 dpi** | **10 dpi** |
| **Immune** | |  |  |  |  |
|  | C4BPA | complement component 4 binding protein, alpha | 0.08 | 0.08 | 0.08 |
|  | CANX | calnexin | 0.06 | 0.06 | 0.06 |
|  | CCL8 | chemokine (C-C motif) ligand 8 | 0.16 | 0.10 | 0.26 |
|  | CD1B | CD1b molecule | 0.25 | 0.28 | 0.29 |
|  | CFB | complement factor B | 0.22 | 0.09 | 0.27 |
|  | CYP11B1 | cytochrome P450, family 11, subfamily B, polypeptide 1 | 0.09 | 0.08 | 0.08 |
|  | HLA-A | major histocompatibility complex, class I, A | 3.27 | 9.48 | 4.72 |
|  | HLA-B | major histocompatibility complex, class I, B | 6.08 | 3.01 | 2.48 |
|  | HLA-DPA1 | major histocompatibility complex, class II, DP alpha 1 | 0.25 | 0.12 | 0.20 |
|  | HLA-DRB1 | major histocompatibility complex, class II, DR beta 1 | 0.16 | 0.33 | 0.27 |
|  | HLA-DRB3 | major histocompatibility complex, class II, DR beta 3 | 3.39 | 13.28 | 0.26 |
|  | HLA-G | HLA class I histocompatibility antigen, alpha chain G-like | 0.20 | 0.22 | 0.26 |
|  | IL17A | interleukin 17A | 3.70 | 5.84 | 16.21 |
|  | IL1R1 | interleukin 1 receptor, type I | 0.33 | 0.31 | 0.27 |
|  | KIR3DL1 | killer cell immunoglobulin-like receptor, three domains, long cytoplasmic tail, 1 | 0.28 | 4.00 | 3.16 |
|  | LILRB3 | leukocyte immunoglobulin-like receptor, subfamily B (with TM and ITIM domains), member 3 | 0.19 | 0.06 | 0.28 |
|  | MS4A2 | membrane-spanning 4-domains, subfamily A, member 2 | 12.86 | 7.70 | 19.61 |
|  | SEMA3A | sema domain, immunoglobulin domain (Ig), short basic domain, secreted, (semaphorin) 3A | 0.12 | 0.11 | 0.21 |
|  | XCL1 | chemokine (C motif) ligand 1 | 8.41 | 3.63 | 4.21 |
|  |  |  |  |  |  |
| **Transcription** | |  |  |  |  |
|  | AR | androgen receptor | 0.18 | 0.17 | 0.24 |
|  | HIST4H4 | histone cluster 4, H4 | 5.24 | 5.06 | 4.76 |
|  | PDX1 | pancreas/duodenum homeobox protein 1-like | 0.18 | 0.22 | 0.21 |
|  | SIX1 | SIX homeobox 1 | 0.08 | 0.07 | 0.07 |
|  | ZNF140 | zinc finger protein 140 | 4.30 | 3.07 | 3.66 |
|  | ZNF323 | zinc finger protein 323 | 3.88 | 5.57 | 4.56 |
|  |  |  |  |  |  |
| **Cell proliferation** | |  |  |  |  |
|  | CASP3 | caspase 3, apoptosis-related cysteine peptidase | 3.03 | 3.58 | 5.29 |
|  | CDC20 | cell division cycle 20 homolog (S. cerevisiae) | 0.03 | 0.03 | 0.08 |
|  | CSF1 | colony stimulating factor 1 (macrophage) | 0.29 | 0.15 | 0.22 |
|  | CYP1A1 | cytochrome P450, family 1, subfamily A, polypeptide 1 | 0.05 | 0.08 | 0.10 |
|  | DNAH1 | dynein, axonemal, heavy chain 1 | 0.10 | 0.12 | 0.27 |
|  | DNM1 | dynamin-1-like | 0.12 | 0.12 | 0.12 |
|  | POLE | polymerase (DNA directed), epsilon | 0.14 | 0.06 | 0.12 |
|  | RB1CC1 | RB1-inducible coiled-coil 1 | 0.13 | 0.12 | 0.11 |
|  | RPS2 | ribosomal protein S2 | 0.09 | 0.22 | 0.15 |
|  | RPS27 | ribosomal protein S27 | 11.99 | 6.74 | 8.11 |
|  | TGM2 | transglutaminase 2 (C polypeptide, protein-glutamine-gamma-glutamyltransferase) | 0.13 | 0.15 | 0.12 |
|  |  |  |  |  |  |
| **Signal transduction** | |  |  |  |  |
|  | CACNA1H | calcium channel, voltage-dependent, T type, alpha 1H subunit | 7.58 | 11.86 | 4.24 |
|  | DCX | doublecortin | 0.33 | 4.15 | 0.28 |
|  | EPHA7 | EPH receptor A7 | 0.16 | 0.19 | 4.12 |
|  | FGF1 | fibroblast growth factor 1 (acidic) | 0.17 | 0.17 | 0.16 |
|  | FGFR1 | fibroblast growth factor receptor 1 | 0.21 | 0.21 | 0.29 |
|  | FGFR3 | fibroblast growth factor receptor 3 | 3.84 | 5.36 | 0.21 |
|  | GNG5 | guanine nucleotide binding protein (G protein), gamma 5 | 0.07 | 0.12 | 0.07 |
|  | GRB2 | growth factor receptor-bound protein 7 | 0.09 | 0.06 | 0.05 |
|  | IGFBP2 | insulin-like growth factor binding protein 2, 36kDa | 6.22 | 3.89 | 1.84 |
|  | ITGB6 | integrin, beta 6 | 0.12 | 0.03 | 0.11 |
|  | KCNIP3 | Kv channel interacting protein 3, calsenilin | 0.18 | 0.23 | 19.82 |
|  | LGR5 | leucine-rich repeat containing G protein-coupled receptor 5 | 0.15 | 0.15 | 0.15 |
|  | NRXN1 | neurexin 1 | 0.33 | 0.19 | 0.13 |
|  | OR2B6 | olfactory receptor, family 2, subfamily B, member 6 | 0.09 | 0.08 | 0.08 |
|  | OR2T2 | olfactory receptor, family 2, subfamily T, member 2 | 0.10 | 0.10 | 0.10 |
|  | OR52E2 | olfactory receptor, family 52, subfamily E, member 2 | 0.02 | 0.02 | 0.02 |
|  | OR56B4 | olfactory receptor, family 56, subfamily B, member 4 | 0.07 | 0.19 | 0.05 |
|  | OR5B21 | olfactory receptor, family 5, subfamily B, member 21 | 0.03 | 0.05 | 0.06 |
|  | SCN2A | sodium channel, voltage-gated, type II, alpha subunit | 0.32 | 7.40 | 6.60 |
|  | SH3GL1 | SH3-domain GRB2-like 1 | 0.04 | 0.09 | 0.13 |
|  | WNT7A | wingless-type MMTV integration site family, member 7A | 0.12 | 0.22 | 0.31 |
|  |  |  |  |  |  |
| **Other** | |  |  |  |  |
|  | ADCY2 | adenylate cyclase 2 (brain) | 0.23 | 0.15 | 0.14 |
|  | ADH1B | alcohol dehydrogenase 1B (class I), alpha polypeptide | 0.22 | 0.20 | 0.28 |
|  | AK7 | adenylate kinase 7 | 0.17 | 0.17 | 0.24 |
|  | ANK1 | ankyrin 1, erythrocytic | 0.18 | 0.22 | 0.22 |
|  | BMP5 | bone morphogenetic protein 5 | 0.13 | 0.13 | 0.29 |
|  | CADM3 | cell adhesion molecule 3 | 0.08 | 0.21 | 0.27 |
|  | CDS1 | CDP-diacylglycerol synthase (phosphatidate cytidylyltransferase) 1 | 0.32 | 0.17 | 0.29 |
|  | CEPT1 | choline/ethanolamine phosphotransferase 1 | 6.35 | 7.42 | 6.26 |
|  | COL6A6 | collagen, type VI, alpha 6 | 0.32 | 0.09 | 0.07 |
|  | CYP2A6 | Cytochrome P450, family 2, subfamily A, polypeptide 6 | 0.27 | 0.18 | 0.31 |
|  | DGKZ | diacylglycerol kinase,zeta | 11.70 | 8.73 | 4.95 |
|  | DMD | dystrophin | 0.13 | 0.08 | 0.12 |
|  | DSE | dermatan sulfate epimerase | 0.26 | 0.16 | 0.21 |
|  | FMO1 | dimethylaniline monooxygenase [N-oxide-forming] 1-like | 0.14 | 0.11 | 0.12 |
|  | GSTA1 | glutathione S-transferase alpha 1 | 0.27 | 0.29 | 0.31 |
|  | KRT17 | keratin 17 | 0.17 | 0.14 | 0.31 |
|  | LRRC4C | leucine rich repeat containing 4C | 0.07 | 18.83 | 0.12 |
|  | MBOAT2 | membrane bound O-acyltransferase domain containing 2 | 0.10 | 0.31 | 0.32 |
|  | MDH1 | malate dehydrogenase 1, NAD (soluble) | 0.19 | 0.19 | 0.18 |
|  | MYL2 | myosin, light chain 2, regulatory, cardiac, slow | 0.03 | 0.03 | 0.04 |
|  | NDST3 | N-deacetylase/N-sulfotransferase (heparan glucosaminyl) 3 | 0.14 | 0.12 | 0.05 |
|  | NKD1 | naked cuticle homolog 1 (Drosophila) | 8.29 | 4.51 | 3.25 |
|  | NLRP1 | NLR family, pyrin domain containing 1 | 0.21 | 0.28 | 0.24 |
|  | NMNAT2 | nicotinamide nucleotide | 0.10 | 0.10 | 0.10 |
|  | PEX5 | peroxisomal biogenesis factor 5 | 0.11 | 0.14 | 0.20 |
|  | SFRP1 | secreted frizzled-related protein 1 | 0.09 | 0.10 | 0.10 |
|  | SLPI | secretory leukocyte peptidase inhibitor | 0.08 | 0.07 | 0.21 |
|  | SNRPD1 | small nuclear ribonucleoprotein D1 polypeptide | 3.73 | 3.33 | 3.06 |
